# Activity of *tjakura* (great desert skinks) at burrows in relation to plant cover and predators

**DOI:** 10.1002/ece3.10391

**Published:** 2023-08-01

**Authors:** Jenna C. H. Ridley, Christine A. Schlesinger

**Affiliations:** ^1^ Research Institute for the Environment and Livelihoods Charles Darwin University Alice Springs Northern Territory Australia; ^2^ Present address: Fenner School of Environment and Society Australian National University Canberra Australia

**Keywords:** activity, burrow, camera trapping, desert, *Liopholis kintorei*, predator

## Abstract

Increased predation where ground cover is reduced after severe wildfire is increasingly implicated as a factor causing decline of vulnerable prey populations. In arid central Australia, one species detrimentally affected by repeated wildfire is the great desert skink or *tjakura* (*Liopholis kintorei*), a distinctive lizard of the central Australian arid zone that constructs and inhabits multi‐entranced communal burrows. We aimed to test whether *tjakura* or predator activity at burrow entrances varied with cover and how *tjakura* respond to predator presence. Using time‐lapse photography, we monitored *tjakura* and predator activity at the largest entrance of 12 burrows ranging from high (>70%) to low (<50%) cover and at multiple entrances of two other burrows. Overall activity did not vary between burrows with high and low cover. Within burrow systems *tjakura* were more active at sparsely vegetated entrances, often sitting wholly or partly inside the burrow. However, consistent between and within burrow systems, skinks spent proportionally more time fully outside where cover was higher. Predators—mostly native—were detected at most burrows, with no apparent relationship between predator activity and cover. Skinks also did not appear to modify their activity in response to predator visits. Our results indicate that *tjakura* may spend more time outside burrow entrances when cover is higher but there was no direct evidence that this related to perceived or real predation risk. Differences in food availability, thermoregulatory opportunities and opportunities for ambush foraging associated with differences in vegetation cover or composition are other factors likely to be important in determining the activity of *tjakura* at burrows. Our research demonstrates the usefulness of camera traps for behavioural studies of ectothermic burrowing animals. The complex relationships between *tjakura* activity and vegetation cover thereby revealed, suggest outcomes of fire‐mediated habitat change on predator–prey interactions are not easily predictable.

## Graphical abstract

Loss of vegetation cover following fire may increase predation risk, even for prey that spend most of their time in burrows. We found *tjakura*, desert‐dwelling communal burrowing skinks, spend more time fully outside their burrow where cover is higher and more time sitting just within burrow entrances where cover is lower. This behavioural response to proximate environmental features may reduce the predation risk for *tjakura* when fire or other disturbances remove cover.

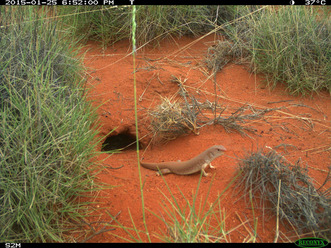

## 1 INTRODUCTION

Fire is a regular disturbance in most vegetation communities of the arid regions of Australia and, if severe, can dramatically reduce cover and diversity of the standing vegetation (Knuckey et al., 2016). These changes often persist for many years and potentially increase vulnerability of many smaller animals to predation if these species rely on cover for shelter (Torre & Díaz, 2004). There has been considerable interest in untangling interactions between fire and predation risk (Doherty et al., 2022), particularly in the fire‐prone ecosystems of the arid and wet‐dry tropical regions of central and northern Australia, where altered fire regimes and introduced predators rate among the most serious threats for native wildlife. There is increasing evidence that the key introduced predators in these regions, cats (*Felis catus*) and the European fox (*Vulpes vulpes*), hunt more effectively in recently burnt areas either because they can navigate more easily or because the refuges of prey species are more visible (McGregor et al., 2014, 2016; Nimmo et al., 2018).

**FIGURE 1:**
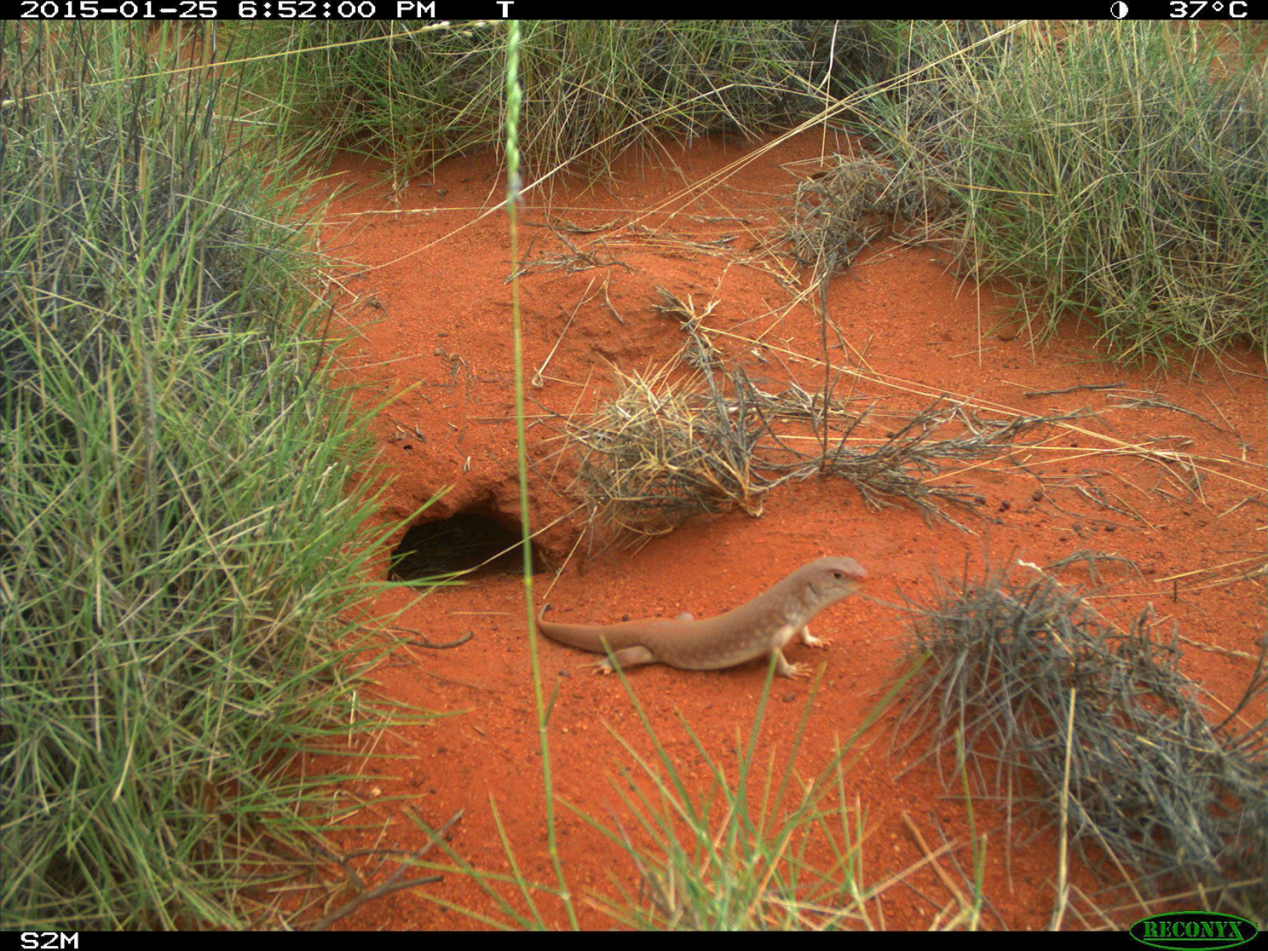
*Tjakura* (great desert skink, *Liopholis kintorei*) outside a burrow entrance.

Our research focussed on a threatened species that inhabits fire‐prone vegetation communities in arid Australia for which such interactions between predation and fire are thought to be important. *Tjakura* (great desert skink; *Liopholis kintorei*) construct and maintain extensive, multi‐tunnelled burrows that become home to adults and their young for many years and breeding seasons (McAlpin, 2001; Figure 1). They exhibit social and cooperative behaviours, and most of their activities are centred around the burrows (McAlpin et al., 2011). Burrows have multiple entrances, some of which have distinctive mounds of excavated soil adjacent to them. There is usually one entrance and mound bigger than the others, with a larger mound which appears to be the most frequently used, however, there has been little documented about the activity of *tjakura* in and around the burrow systems. *Tjakura* are omnivorous, consuming plant material and invertebrates, with termites (Order: Blattodea, Superfamily: Termitoidea) thought to be the most important prey item (Chapple, 2003; McAlpin et al., 2011). They are thought to exhibit predominantly ambush strategies to catch prey, sitting partially within or near their burrow entrance and opportunistically taking invertebrates as they pass by, and only infrequently moving away from the burrow entrance to forage actively at night (Chapple, 2003; Department of Climate Change, Energy, the Environment and Water, 2023). *Tjakura* have also been observed thermoregulating using variable postures and basking while half in the burrow entrance (Chapple, 2003). Wildfire and introduced predators are recognized as key threats for the species (Moore et al., 2015; Moore, Kearney, et al., 2018).

One of the known strong‐hold populations of the great desert skink is in the Uluru‐Kata Tjuta National Park in central Australia (McAlpin, 2001) where they are known as *tjakura* which is their Pitjantjatjara and Yankunytjatjara name (Indigenous Desert Alliance, 2022) and the name we use here. Pitjantjatjara is the most common language spoken by Anangu, the local Aboriginal custodians where our research took place. The species is also known by other names in other central Australian Aboriginal languages and regions including *warrarna* (Warlpiri) and *mulyamiji* (Muanyjilijarra, spoken by Martu people), *tjalapa* (Pintupi) and *nampu* (Aran, spoken by Anmatjere) (Indigenous Desert Alliance, 2022). Genetic research by Dennison et al. (2015) demonstrated that skink haplotypes at Uluru were unique to the area and that overall there is very little connectivity between the six known stronghold localities for the species (Sangsters Bore, Newhaven, Watarru, Docker River and Warburton). Of the skinks sampled, those from Uluru were the most genetically distinctive, leading to the recommendation that the population should be considered its own delineation for the purpose of conservation management (Dennison et al., 2015). At the Uluru site, *tjakura* burrows tend to occur in areas with slightly higher vegetation cover compared to the surrounding landscape and often inhabit areas with sparsely scattered shrubs (e.g. *Grevillea eriostachya*) (Ridley et al., 2018) but it is not known whether this relates to shelter, increased abundance of invertebrate prey (Morton & James, 1988) or other structural habitat features. The risk of predation for *tjakura* is probably highest when they are outside their burrows, with burrows providing a refuge. However, burrow sites may also be a means by which predators locate or regularly relocate skink colonies (Moore et al., 2015). As a likely focal point for predator–skink interactions burrow entrances provide an excellent opportunity to study the behaviour of *tjakura* in relation to vegetation cover and predator activity.

Based on previous research suggesting increased cover is likely to favour *tjakura* through limiting the indirect effect of predation pressure (Moore et al., 2015), we hypothesized that;

1. *tjakura* would be more active at burrows that had higher vegetation cover,
2. predator activity would be less at burrows with greater vegetation cover due to decreased visibility of the *tjakura* and their burrow systems,
3. *tjakura* activity at the burrow entrance would decrease after a predator visit, because either the predation attempt was successful or, in the short term, skinks modified their behaviour in response to a greater perceived threat of predation.

We also expected that there may be an interaction between diel activity patterns and vegetation cover, especially in relation to activity in daylight compared to dark. To gain a better understanding of how *tjakura* use different entrances within a burrow system, we also monitored all the entrances of two burrows in relation to specific attributes of the entrance, including vegetation cover.

## 2 MATERIALS AND METHODS

This study was conducted within Uluru‐Kata Tjuta National Park, Northern Territory, in sand plain habitat (25.3009° S, 130.7184° E), over one active season. The *tjakura* population is restricted to the sand plain areas of the park which are dominated by spinifex (*Triodia* spp.) and tussock grasses with a very sparse over‐storey of honey grevillea (*Grevillea eriostachya*) and desert oaks (*Allocasuarina decaisneana*) and contains a mosaic of vegetation with different fire histories.

### 2.1 Burrow selection

Twelve active *tjakura* burrows were chosen for monitoring out of a larger sample of 20 burrows that were being actively used by skinks at the time of our study. The 20 burrows were a subset of burrows that had previously been mapped and regularly monitored by the National Park rangers (Ridley et al., 2018). To select burrows for monitoring, all known burrows were checked for signs of recent activity and habitation, indicated by the presence of fresh scats and tracks. For the 20 burrows identified as active the percentage projected horizontal canopy cover of all plant species within a 2.5 m radius circle centred at the largest burrow entrance was visually estimated.

We then selected 12 of these burrows to represent two distinct cover categories (low/high) for monitoring. The six burrows with the lowest vegetation cover (all <50%) around the main entrance were classed as low cover. The six burrows with the highest vegetation cover (all >70%) were classed as high cover. The three burrows with the lowest cover (26%–36%) were in areas that had been burnt 3 years previously in a 2012 wildfire, and the other three low cover burrows (41%–49% cover) had not been burnt for 12 years. High cover burrows were all in areas unburnt for at least 12 years. Burrows varied in size and number of entrances (Table 1). Burrow size was estimated by measuring the distance between entrances furthest away from each other in two perpendicular directions and multiplying to derive an estimated area.

**TABLE 1:** Characteristics of the 12 burrows chosen for monitoring, ordered from lowest to highest vegetation cover and categorized as having low cover (L1‐L6) or high cover (H1‐H6).

Burrow #	Vegetation cover (%)	Years since burnt	Burrow size (m^2^)	Number of entrances
L1	26	2	10	8
L2	30	2	10.5	5
L3	36	2	12	6
L4	41	12	9	7
L5	49	12	25	10
L6	49	12	50	17
H1	70	12	22.5	10
H2	70	12	12	9
H3	73	12	42	7
H4	74	12	60	12
H5	74	12	20	16
H6	80	12	14	8

### 2.2 Monitoring *tjakura* activity

*Tjakura* activity was monitored using 10 Reconyx HC600 and 2 Reconyx HC800 cameras. One camera was set up at each burrow at what appeared to be the main entrance. Every burrow had multiple entrances and the main entrance was determined as the biggest one associated with the biggest soil mound (a distinctive feature at burrow entrances created from excavated soil), where this was obvious. Another distinctive characteristic of *tjakura* burrows is the presence of a ‘latrine’ or scat pile which is consistently used as a defecation site by burrow inhabitants. If there were two entrances and mounds of similar size within a burrow system, the entrance closest to the latrine or most centrally located among the other entrances was chosen.

Cameras were installed 1 m from the burrow entrance, mounted on platforms angled downwards at 60° to the ground and screwed onto metal garden stakes, 60 cm above the ground. This setup produced a 1 m^2^ field of view centred on the burrow entrance. A small plywood shelter, attached directly above each camera, shaded it from the extreme heat typical of the central Australian summer.

Cameras were set to the time‐lapse mode to take one photo every minute for two 18‐day periods in early summer (from 12:00 midday 27 November to 12:00 midday 15 December 2014), and mid‐late summer (from 12:00 midday 24 January to 12:00 midday 11 February 2015). Batteries and SD cards in cameras were replaced every 5–6 days, but battery failure led to the loss of 8 h of data from one burrow and 12 h each from two other burrows.

We used the number of images with *tjakura* visible as an index of their activity at the burrow entrance. Although we refer to these periods as ‘active’, most of the time when lizards were partially or fully emerged, they remained quite stationary (i.e. were in the same place in multiple consecutive images). When there were continuous periods (i.e. consecutive images) with *tjakura* present, ‘active bouts’ was used to describe this activity. When there were no *tjakura* visible on an image, we assumed the residents had either retreated inside the burrow or were actively foraging (or engaging in another activity) away from the burrow entrance.

### 2.3 Data analysis

**FIGURE 2:**
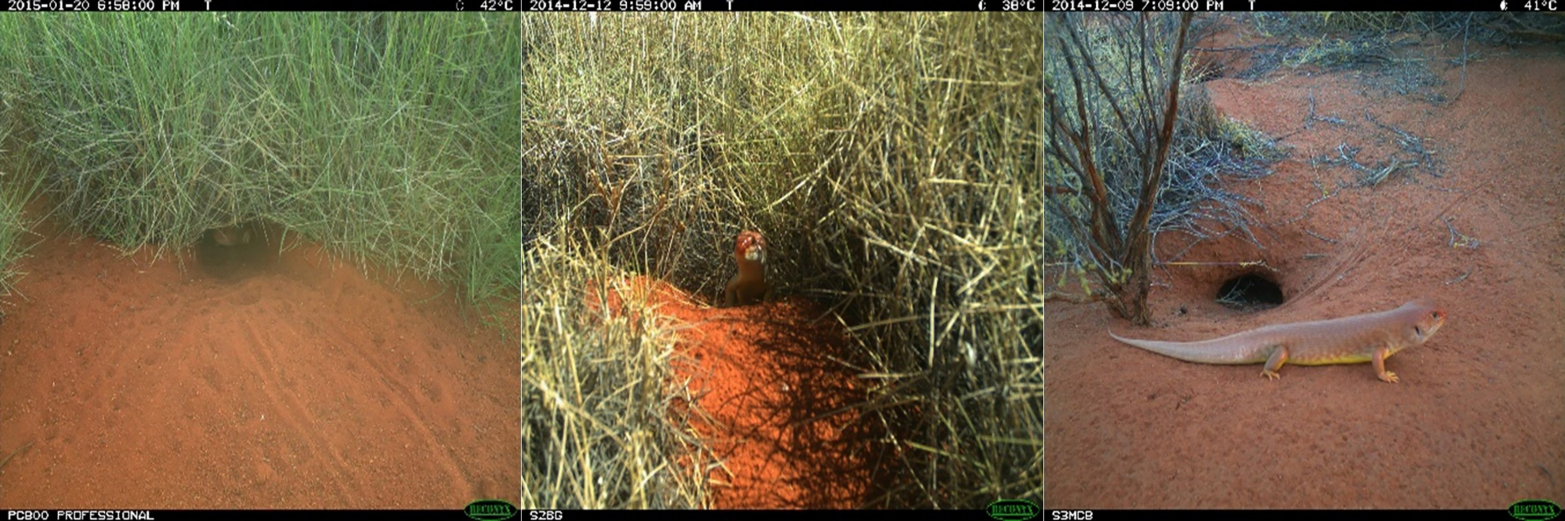
Positions of *tjakura* at the main burrow entrance, coded as, from left to right; in the burrow, half in the burrow, and outside the burrow (counted as outside when front and back legs were visible outside of the entrance).

The images were initially sorted into three categories: one or more *tjakura* visible, other vertebrate species visible or no vertebrate visible. The images with skinks present were then divided into three position categories: fully inside the burrow entrance, half emerged with front legs visible or fully emerged from the burrow entrance, with hind legs visible outside (Figure 2). There were three clearly distinct size classes of *tjakura* in the images including very small, medium and large or adult size, so we were able to assign individuals into three approximate age classes; juvenile (current year hatchlings), sub‐adult or adult. Where there were two or more *tjakura* in the image, each was classified separately for size and position.

We were not always able to distinguish among different individuals from the same burrow. However, obvious differences in size, colour, the presence of distinguishing features (e.g. part of the tail missing) or the presence of multiple individuals in single images allowed us to determine a minimum number of individuals present at each burrow during each recording period.

### 2.4 Activity at burrows in relation to burrow characteristics and vegetation

Repeated measures analysis of variance was used to determine whether the minimum number of individuals varied between the two recording periods (within subjects effect) or between burrows with high and low vegetation cover. We then used Pearson correlations to test how *tjakura* activity at the main burrow entrance related to the minimum number of lizards occupying the burrow, the number of entrances in the burrow system and vegetation cover at the burrow.

### 2.5 Response by *tjakura* to predator activity at burrows

All vertebrates known or suspected to be predators of *tjakura* were included in analyses. The number of discrete ‘visits’ by predators to burrows was determined based on the sequence of images. If the same predator was visible in consecutive images, this was counted as a single visit. We then tested for correlations between vegetation cover at burrows and the number of predator visits.

To determine whether *tjakura* reduced their activity in response to predator visits, graphs were produced to show the timing of their active bouts in relation to predator visits. The activity of skinks before and after predator visits was compared based on (a) the time interval (min) between a *tjakura* being visible and the arrival of a predator and the interval (min) between a predator leaving and a *tjakura* becoming visible again, and (b) the number of active bouts by *tjakura* in the 24 h prior to predator arrival and post‐predator departure. The null hypothesis (no effect of predators) was that there would be no difference in the 24 h before and after a predator visit in the time interval between *tjakura* activity and predator activity or the number of *tjakura* active bouts. Time data were log transformed prior to analyses.

### 2.6 Daily activity patterns of *tjakura*

To investigate patterns in daily activity we plotted the time *tjakura* spent outside the burrow against the average activity of *tjakura* in the early and late active season. The early active season was considered as the time between *tjakura* first emerging during spring until end of December and the late active season as roughly January to March. We then split the data into dawn, day, dusk and night. Dawn and dusk were defined as an hour‐long period during which sunrise and sunset occurred. In late 2014 dawn fell approximately between 05:00 and 06:00 (sunrise range 5:47 a.m.–5:49 a.m.) and in early 2015 dawn fell between 06:00 and 07:00 (sunrise range: 6:15 a.m.–6:58 a.m.), and dusk in both years was approximately between 19:00 and 20:00 (sunset November/December range: 7:19 p.m.–7:32 p.m., January/February: 7:39 p.m.–7:31 p.m.) (Australian Government Bureau of Meteorology, http://www.bom.gov.au, accessed July 2021). We used a three‐factor repeated measures ANOVA with time (dawn/day/dusk/night) and vegetation (low/high) as the fixed factors and burrow site as a random factor nested within vegetation and Tukey's post‐hoc test was used for pair‐wise comparisons. To do this, we converted number of images to images per hour to account for the different lengths of each period.

### 2.7 *Tjakura* activity at multiple entrances within a burrow system

Two additional burrows were selected from the remaining eight of the 20 active burrows; one (burrow A) with four entrances and one (burrow B) with seven entrances. Reconyx cameras were set up at every burrow entrance in the same way as described previously. Width, height, opening, diameter and height (cm) of the mound, as well as percentage cover of vegetation in a 1 m^2^ quadrat centred over each entrance were measured and the main entrance was identified, according to previous protocols.

Activity at burrow entrances was recorded at all entrances simultaneously for 4 days in early February 2015. Cameras were set to time lapse taking one picture per minute. One camera (at burrow A, Entrance 1) malfunctioned, and that entrance was excluded from all analyses. We used correlations to test whether the level of activity at burrow entrances was associated with burrow size, minimum number of individuals using the system, overall activity and vegetation cover at each entrance.

## 3 RESULTS

### 3.1 Activity at burrows

There was no difference in the number of entrances of burrow systems with low vegetation cover (mean 8.8; range 8–17) and high vegetation cover (mean 10.3; range 7–16; *F*_1,10_ = 0.46; *p* = .52). There were at least one to six different individuals in each burrow during each survey period (Table 2). At least two age classes and two individuals could be confirmed as inhabiting all but one burrow. There was no relationship between the number of burrow entrances and the minimum number of *tjakura* recorded in each burrow system, either in the early (Pearson's correlation: *r* = .20; *p* = .54), or mid‐late summer (*r* = .33; *p* = .29) survey period. There was, however, a significant effect of time on the minimum number of lizards recorded per burrow (repeated measures ANOVA: *F*_1,10_ = 5.77; *p* = .037), with more individuals detected in the second survey period (Table 2). There was no effect of vegetation density (*F*_1,10_ = 0.06; *p* = .81), nor any time × vegetation density interaction (*F*_1,10_ = 0.47; *p* = .51) on the minimum number of individuals at the burrow.

**TABLE 2:** The number of *tjakura* (mean, standard error and range) detected at burrows with low and high vegetation cover each summer.

Vegetation cover	Minimum number of *tjakura*: Mean (SE), range	
	2014	2015
Low	3.0 (0.37) 2–4	4.5 (0.76) 1–6
High	3.2 (0.60) 2–5	4.0 (0.52) 2–6

The was no correlation between the number of entrances to the burrow system and the total number of recorded images with lizards at the main entrance. However, there was a positive relationship between the total number of images with *tjakura* and the minimum number of individuals detected (*r* = .59, *p* = .042) at a burrow. More individuals resulted in more records of activity. Subsequent analyses using either absolute measures of activity, or activity per individual *tjakura* showed identical trends, so we only report absolute values here.

There were no significant correlations between vegetation cover and the total number of *tjakura* images or images of *tjakura* inside or outside the burrow, however, the proportion of time *tjakura* spent outside of the burrow was positively correlated with higher vegetation cover at the burrow site (*r* = .637, *p* = .026).

### 3.2 Response by *tjakura* to predator activity at burrows

**FIGURE 3:**
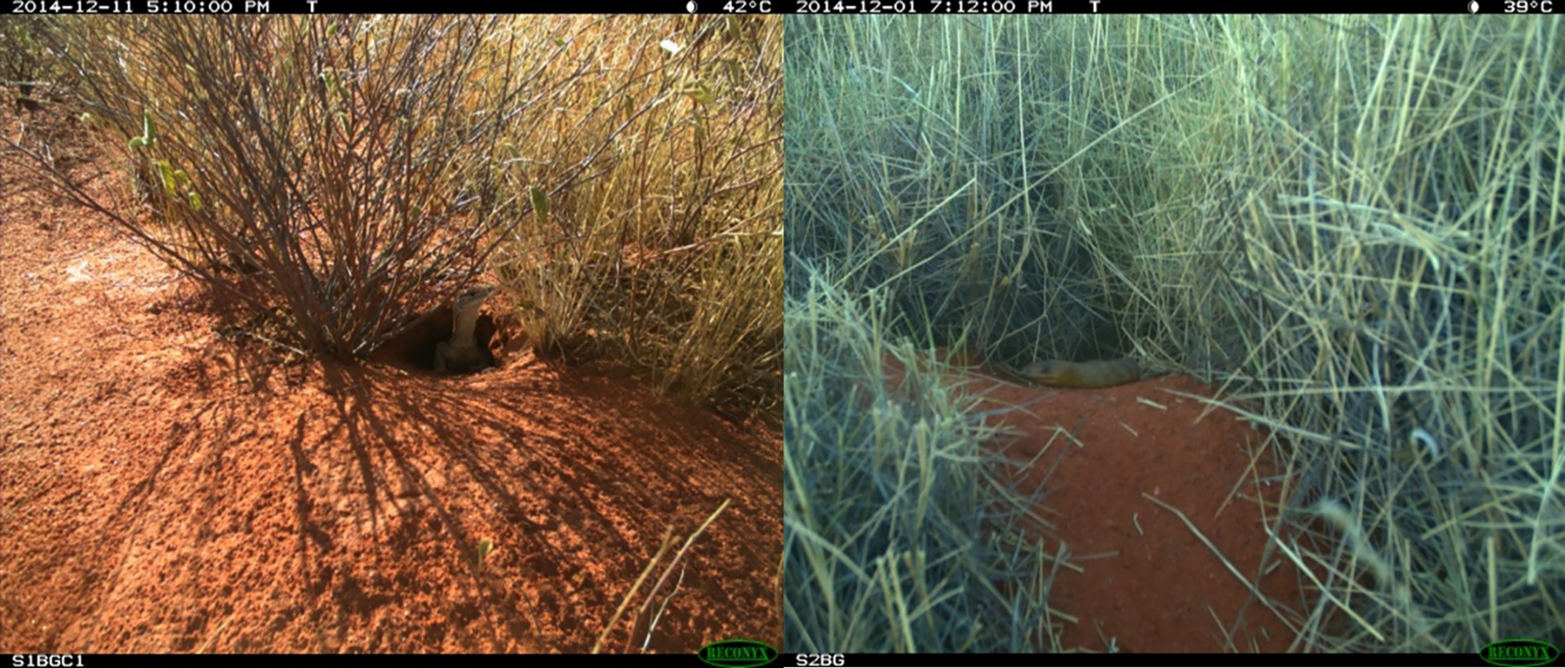
Predatory species recorded at *tjakura* burrows included sand goannas and woma pythons.

Four predator species and several unidentified species were detected at the burrow entrances. The most common predator species observed was the sand goanna (*tinka* in Pitjantjatjara; *Varanus gouldii*; 30 visits across all burrows), followed by the mulgara (*murtja* in Pitjantjatjara; *Dasycercus blythi*; eight visits across all burrows) and woma python (*kuniya* in Pitjantjatjara; *Aspidites ramsayi*; eight visits across all burrows) (Figure 3). The European fox (*Vulpes vulpes*) was only detected twice and was the only introduced predator observed.

Predators were recorded at every burrow expect for H3 and H5, two of the high cover burrows. Despite this, overall there was no significant correlation between the number of predator visits at low and high vegetation cover burrows (*r* = −.322, *p* = .307). The same predators were detected at low and high cover burrows, except the fox which was only detected at low cover burrows.

**FIGURE 4:**
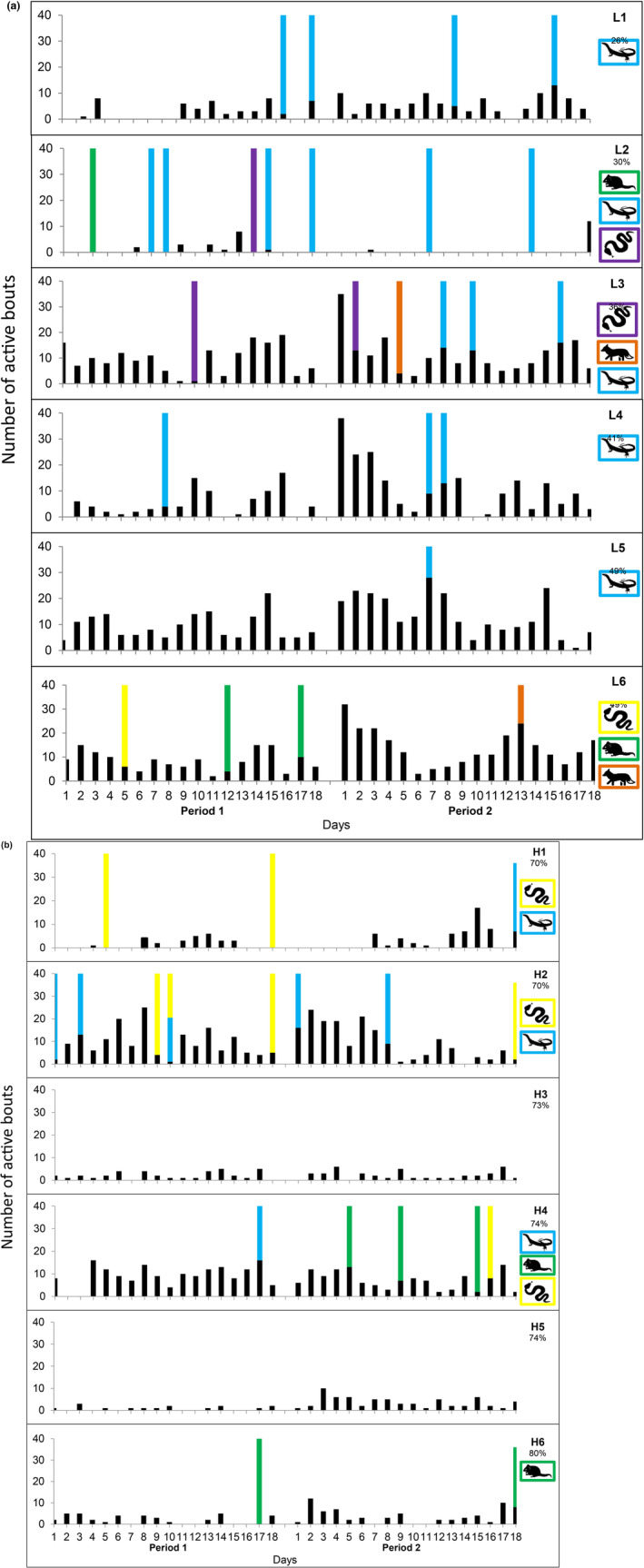
(a) Daily activity (# active bouts) of *tjakura* at the six burrows with low cover (percent cover indicated on each graph) from two 18‐day periods of camera monitoring. Predator visits over this time are displayed by coloured bars. Goannas = blue, woma pythons = yellow, mulgara = green, fox = orange and unidentified snake = purple. (b) Daily activity (# active bouts) of *tjakura* at the six burrows with high cover (percent cover indicated on each graph) from two 18‐day periods of camera monitoring. Predator visits over this time are displayed by coloured bars. Goannas = blue, woma pythons = yellow and mulgara = green.

Activity of *tjakura* was highly variable between the 12 burrow systems and there was also much variation between the number and species of predators visiting each of the burrows (Figure 4a,b). There was no obvious decline in *tjakura* activity following predator visits at burrows except for burrow L2 and possibly H2. *Tjakura* activity at burrow L2 was minimal throughout the monitoring period and by the late active season almost no activity was detected until the last day of monitoring (Figure 4a). Predator presence at L2 was high in the early season and successful predation on some individuals occupying this burrow could account for the observed decline in activity over time. Burrow H2 was visited by a variety of predators, and after the last recording of a sand goanna there was a distinct decline in *tjakura* activity (Figure 4b).

Analyses were conducted on all predators grouped together because most predatory species were detected at burrows too infrequently for independent analysis of their impact on *tjakura* activity. Sand goannas were the exception and were therefore analysed separately. Comparing activity before and after a visit, we did not detect any significant impact of predator visits (all predators or goannas) on the length or the number of active bouts (Table 3). There also were no significant interactions between time and vegetation, suggesting the response (or lack of response) to predators did not differ between burrows with high or low vegetation cover. *Tjakura* activity differed significantly among burrows but this did not appear to relate to predator activity or vegetation cover (Table 3).

**TABLE 3:** Results from three‐factor repeated measures ANOVA testing for differences between *tjakura* activity (measured as log of minutes active and the number of active bouts) 24 h before and after a predatory visit (all predators and goannas) at burrow entrances with low and high vegetation cover; <.05*, <.01**, <.001***, NS, no significance.

Test	Source	Df	MS	*F*‐value	*p*‐Value
All predators (log minutes)	Time	1	0.327	1.46	.236 (NS)
	Vegetation	1	0.815	0.30	.599 (NS)
	Burrow (veg)	8	3.800	25.91	<.001***
	Time x veg	1	0.00977	0.04	.836 (NS)
	Burrow x time (veg)	8	0.146	0.37	.934 (NS)
All predators (*tjakura* active bouts)	Time	1	8.705	0.27	.608 (NS)
	Vegetation	1	234.812	1.28	.288 (NS)
	Burrow (veg)	8	250.308	8.37	.003**
	Time x veg	1	0.243	0.01	.932 (NS)
	Burrow x time (veg)	8	29.891	0.81	.596 (NS)
Sand goannas (log minutes)	Time	1	0.236	1.83	.186 (NS)
	Vegetation	1	0.00184	0.00	.968 (NS)
	Burrow (veg)	6	1.533	19.25	.001**
	Time x veg	1	0.0277	0.22	.646 (NS)
	Burrow x time (veg)	6	0.0796	0.38	.890 (NS)
Sand goannas (*tjakura* active bouts)	Time	1	36.510	0.63	.451 (NS)
	Vegetation	1	0.131	0.00	.978 (NS)
	Burrow (veg)	6	251.725	3.07	.099 (NS)
	Time x veg	1	102.432	1.77	.222 (NS)
	Burrow x time (veg)	6	81.909	4.73	.001*

### 3.3 Daily activity patterns of *tjakura*

**FIGURE 5:**
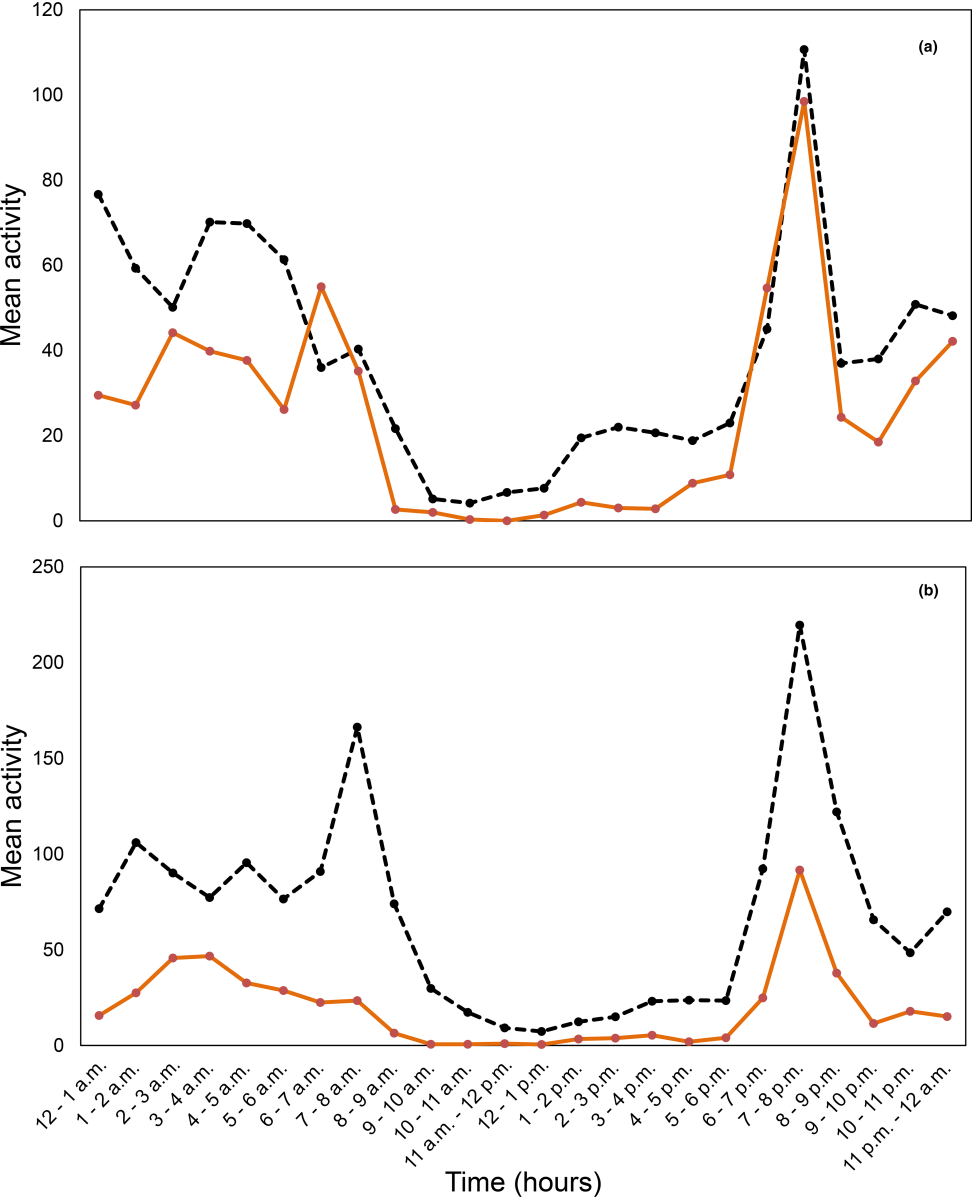
Mean hourly activity (total images) at burrows with low (dashed black line) and high (solid orange line) vegetation cover in (a) 2014 and (b) 2015. Standard errors are omitted to simplify the figure and enable comparison of the timing of activity at high and low cover burrows.

Diel activity of *tjakura* was characterized by low activity during the middle of the day, and peak activity in the dawn and dusk crepuscular periods. This pattern was consistent between low and high vegetation cover burrows (Figure 5). On average, using total images (i.e. *tjakura* visible either inside or outside the burrow) as an index of activity, *tjakura* in burrow systems with low vegetation cover were more active, with this activity more obvious during the late active season. Average activity at burrows with high cover did not increase noticeably between the early and late active periods whereas activity at burrows with low cover was notably higher in the late active season (Figure 5).

**FIGURE 6:**
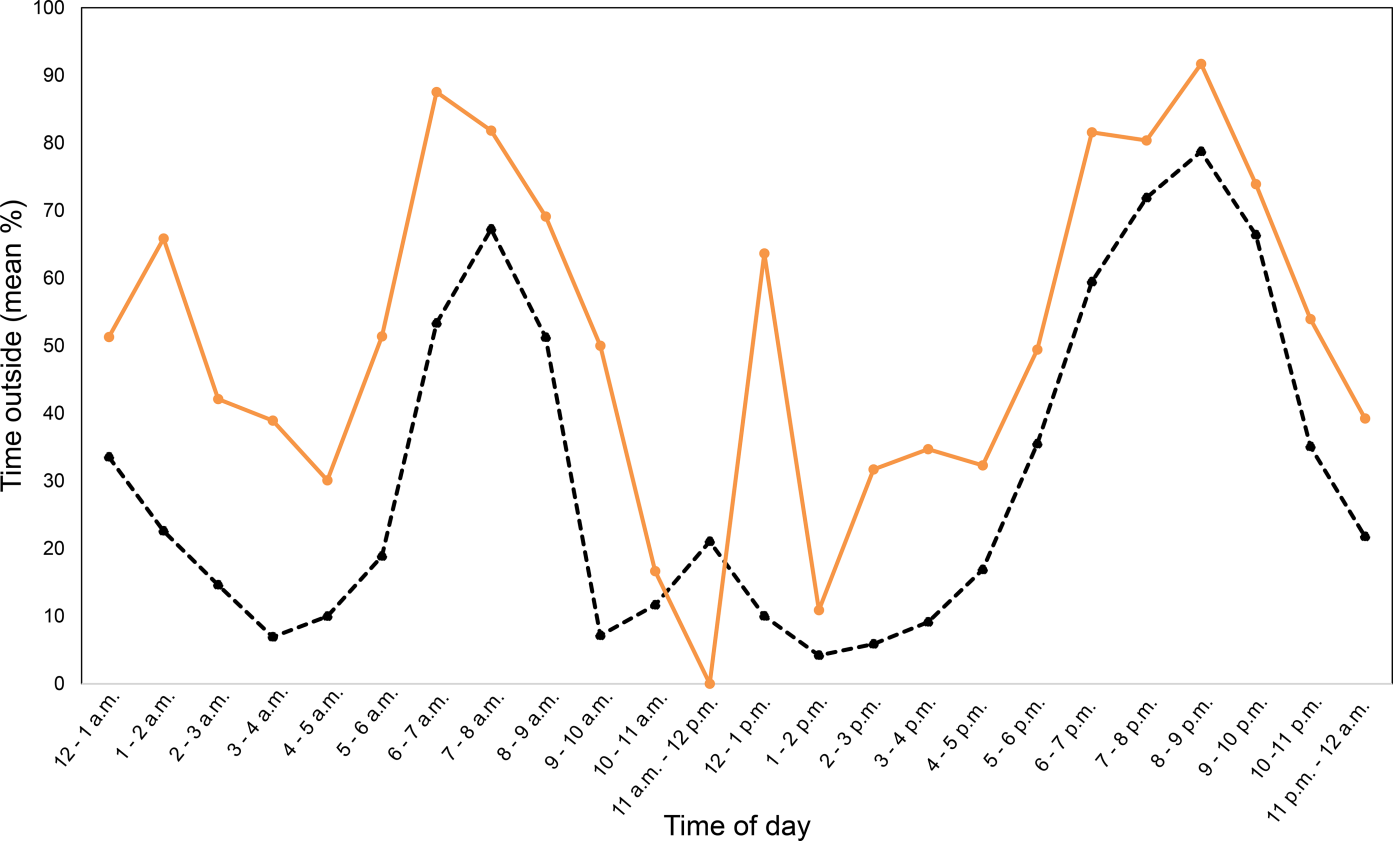
Percentage of time per hour *tjakura* spend outside of the main burrow entrance over 36 days in low (black dashed line) and high (orange solid line) vegetation cover.

*Tjakura* spent the most time outside burrows 2–3 h after dawn as well as just before and in the 3 h after dusk (Figure 6). Although also frequently observed at the burrow entrance in the early morning before dawn (Figure 6) *tjakura* tended to be inside the entrance rather than active at the surface at this time. Time *tjakura* spent outside the burrow was 20% greater on average at burrows with high cover for most times of the day, however, variation between burrows was high and this was not a significant effect in any analyses.

**FIGURE 7:**
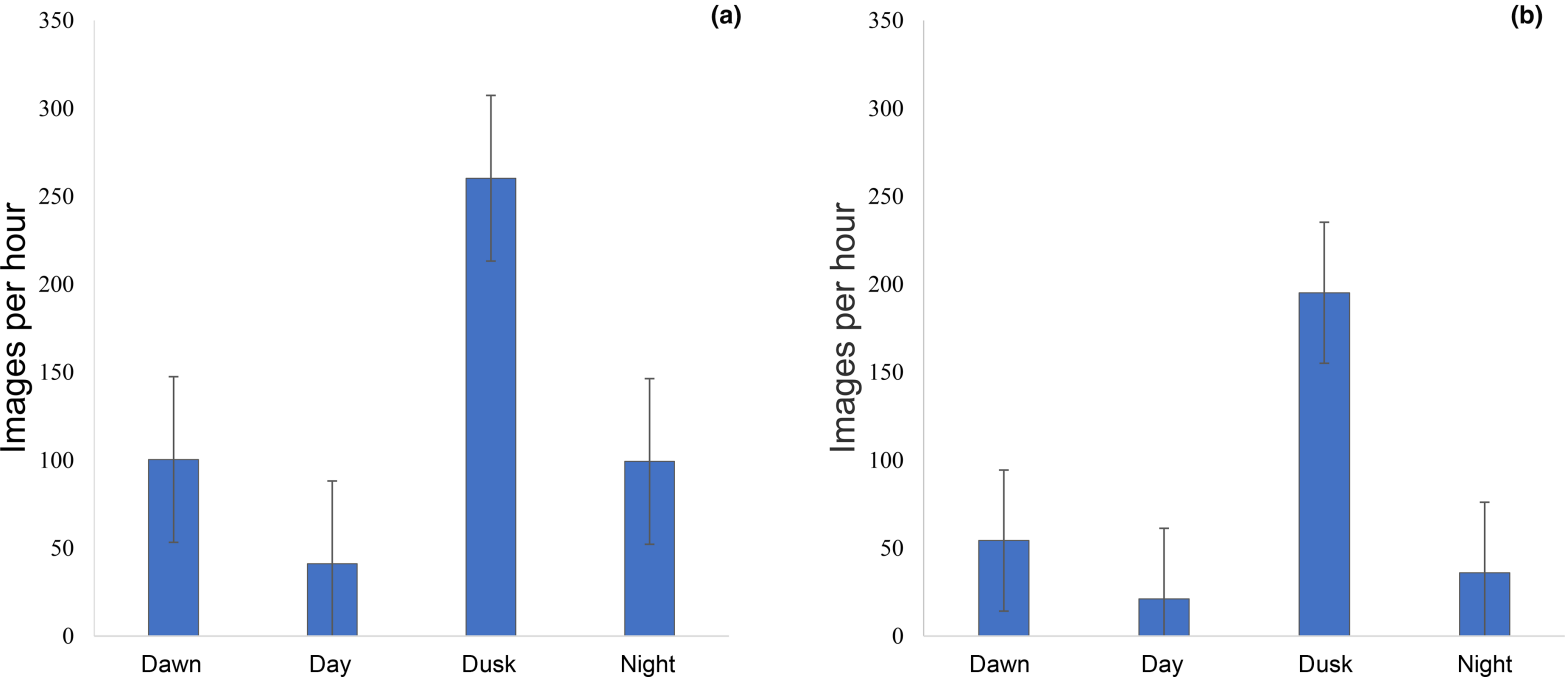
*Tjakura* activity (mean images ±SE per hour) during dawn, day, dusk and night; (a) is total images with *tjakura* visible at the entrance and (b) images where the *tjakura* is outside the burrow entrance.

Time of day significantly affected total images per hour (RMANOVA: df = 3,3, *F* = 8.05, *p* < .001) and outside images per hour (RMANOVA: df = 3,3, *F* = 12.23, *p* < .001) and the post‐hoc tests showed total images and outside images differed significantly between day (when *tjakura* were least active) and dusk (when *tjakura* were most active) (total images: *t* = 7.449, *p* < .05, outside images: *t* = 7.020, *p* < .05). *Tjakura* were moderately active at dawn and night, but activity (total and outside images) was highly variable at these times and did not differ significantly from dusk or day (Figure 7). However, when accounting for the number of individuals at a burrow (by dividing the number of outside images by the minimum known number of individuals), the higher activity at dusk compared to all other times was significant (df = 3, *T* (dawn/dusk) = 7.183, *T* (day/dusk) = 8.004, *T* (night/dusk) = 8.862, *p* < .05).

### 3.4 *Tjakura* activity at different entrances within burrow systems

There was a large amount of variation in vegetation cover and activity at different entrances within a burrow (Table 4). All entrances were used by multiple individuals and, somewhat unexpectedly, the main entrance of burrow B, identified prior to monitoring, had the lowest activity (total images) compared to other entrances at that burrow. Also, at least two juveniles and two sub‐adults were detected at burrow B, but only one individual of each subclass was detected at the main entrance. At burrow B there was no significant correlation between entrance diameter or mound height and *tjakura* activity.

**TABLE 4:** Minimum number of individuals, age classes (j = juvenile, sa = sub‐adult and a = adult) and total images at each entrance of burrow A (A2–A4) and burrow B (B1–B7) (Entrance A1 camera malfunctioned).

Entrance	Vegetation cover (%)	Minimum # individuals detected at entrance	Age classes	Total images
A2—MAIN	0	5	1j, 2sa, 2a	910
A3	23	4	1j, 2sa, 1a	809
A4	52	3	1j, 1sa, 1a	37
B1	20	3	2j, 2sa, 1a	671
B2	48	3	1j, 1sa, 1a	285
B3—MAIN	65	3	1j, 1sa, 1a	40
B4	15	3	1j, 1sa, 1a	1187
B5	61	3	2sa, 1a	63
B6	55	4	2j, 1sa, 1a	234
B7	55	3	1j, 1sa, 1a	132

**FIGURE 8:**
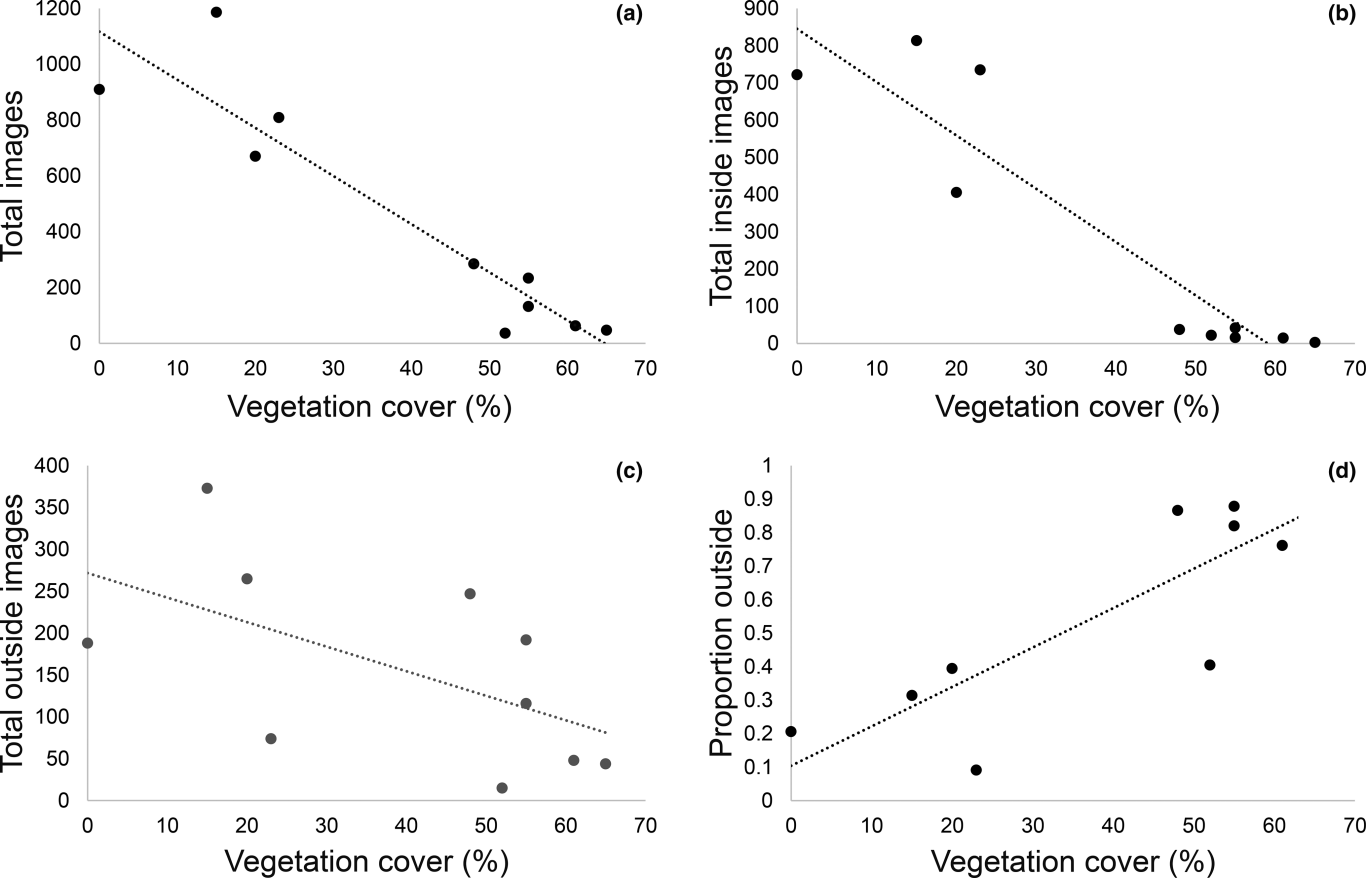
Correlations between vegetation cover at burrow entrances and (a) total *tjakura* images, (b) total number images with *tjakura* inside the burrow, (c) total number of images with *tjakura* outside the burrow and (d) proportion of time *tjakura* are outside (ns).

Overall, activity at the burrow entrance (total images) was strongly negatively correlated with vegetation cover (*r* = −.932, *p* < .001), with most activity detected at entrances with less cover (Figure 8a). However, when *tjakura* were active (either inside or outside the entrance), the proportion of time spent outside was positively correlated with vegetation cover (*r* = .845, *p* = .002; Figure 8b). Consistent with this trend, there was also a negative relationship between total images inside the burrow and vegetation cover (*r* = −.928, *p* < .001; Figure 8c). These results show that *tjakura* were more active, in or near entrances, where vegetation cover was low but were more likely to be wholly outside the entrance when they were active at high cover entrances.

## 4 DISCUSSION

*Tjakura* have several known native and introduced predators including feral cats, foxes, dingoes, sand goannas and several species of snake, although the level of threat posed by different predators is not well understood. Feral cats have been identified as the most significant predator of the *tjakura* population at Newhaven Wildlife Sanctuary (Warlpiri Country), approximately 350 km north of Uluru, based on predator scat contents and the volume of predator tracks near burrows (Moore, Kearney, et al., 2018). Cats may be able to locate skink burrows more easily in open areas where they are more visible and, once a colony has been located, the clustered distribution of burrows within a colony increases their vulnerability to detection and repeat visits (Moore et al., 2015). Foxes and cats have been observed sitting at burrows, waiting for the skinks to emerge (Chapple, 2003); however, there was no evidence of this behaviour during our study. Surprisingly, we detected no feral cats at the burrows we surveyed. We also recorded only two single images of foxes (each at a different time and burrow location) which suggests the foxes spent less than a minute at the monitored burrow entrance on these occasions, although they may have moved to other entrances or remained in the vicinity of the burrow, but out of the field of view of the camera.

It was unclear whether the three native predator species detected at burrows were actively seeking to prey on the *tjakura* or whether they were successful because time‐lapse photography did not provide a continuous record of activity. In contrast to foxes, sand goannas and woma pythons were occasionally present for more than 10 min with one sand goanna visible at a burrow for 60 consecutive minutes. On some occasions goannas and pythons were recorded entering or exiting the burrow, strongly suggesting encounters that may have led to predation. We also captured multiple consecutive images of sand goannas sitting at the burrow entrance with front legs splayed and ventral surface of body in contact with the ground, exhibiting what appeared to be thermoregulatory behaviour, as well as sitting just inside the burrow (see Figure 3). This suggests they may have been using the burrow as shelter (Indigenous Desert Alliance, 2022). Woma pythons are typically ground dwelling snakes that require thermally buffered shelters. Unable to dig burrows themselves, they have been recorded as spending 69% of their refuge time sheltering within underground burrows created by other species (Bruton et al., 2014). Their use of *tjakura* burrows for shelter may account for at least some of the multiple records of pythons at burrow entrances, although they undoubtably pose a risk to the resident *tjakura* while there. Moore, Kearney, et al. (2018) also found large snakes to be commonly active near burrows at Newhaven and directly observed predation of a *tjakura* by a woma python (Moore, Kearney, et al., 2018). Mulgara, medium‐sized dasyurid predators, have also been known to use *tjakura* burrows for shelter (McAlpin, 2001). While capable of preying on *tjakura*, there is limited evidence to suggest that mulgara are major predators of the species.

The interaction between predation and fire—where skinks in open habitat are more exposed to the threat of predation—is thought to pose the greatest threat to *tjakura* populations (McAlpin, 2001; McGregor et al., 2014; Wilson, 2014). However, our hypothesis that predator activity would be greater at burrows with lower cover, was not supported. We also found no conclusive evidence that *tjakura* altered their level of activity at the burrow entrance following a visit by a predator, although low activity at two burrows after predators were detected suggests that successful predation may have occurred in these instances. The relatively low number of predator visits to the monitored burrows overall limited our ability to test for these effects.

Only limited evidence from our study supports the hypothesis that *tjakura* are disadvantaged by low cover. In fact, *tjakura* were observed at the surface, albeit partially or fully within the burrow, more frequently at burrows with low cover. However, the proportion of time *tjakura* spent outside at burrows with low cover was significantly less than at high cover burrows, both between and within burrows. Pygmy blue‐tongue lizards (*Tiliqua adelaidensis*), exhibit somewhat comparable behaviour, spending less time foraging and basking in burnt areas (Fenner & Bull, 2007). The tendency to spend less time exposed in open areas may be linked to an increased risk of predation and aligns with suggestions that *tjakura* burrows in wildfire affected areas are frequently abandoned (Wilson, 2014). Open areas may, however, provide better opportunities for skinks to ambush prey from within the burrow entrance, where they are concealed from both prey and predators, and potentially better opportunities for basking while partially or fully within the burrow, as discussed further below.

### 4.1 General natural history and behavioural observations

Prior to our study, annual surveys of *tjakura* burrows at Uluru‐Kata Tjuta National Park were regularly completed during late summer (February/March), when *tjakura* appear to be most active and burrows are most visible due to accumulated activity over the active season. Activity commences in spring, around September or October, and in the Newhaven population mating activity has been recorded in September with females giving birth approximately 10 or 11 weeks later (Moore et al., 2015). This is broadly consistent with our observations of juveniles from the beginning of the late November survey. We recorded increased numbers of individuals and higher activity at the burrow entrances in the late active season whereas the minimum number of identifiable juvenile lizards only increased at two burrows, so our results seem to reflect mainly a seasonal increase in activity, previously documented, rather than new recruitment. Because juveniles are much smaller and mostly active during the night, they tend to also be less visible which may also make it more difficult to confirm their presence. During the late active season many more images had multiple individuals present together, often of similar size, which enabled us to positively confirm that there were at least two or three sub‐adults present at individual burrows.

*Tjakura* have previously been described as predominantly nocturnal (Chapple, 2003), however our data, consistent with other more recent work (Moore, Stow, & Kearney, 2018), shows primarily crepuscular activity. Activity was consistently lowest during the middle of the day, and highest around dusk and this did not vary with respect to vegetation cover. We observed some interesting behaviour, such as *tjakura* sitting in the burrow entrance, sometimes for hours at a time, often in the pre‐dawn period. This may be a thermoregulatory strategy, documented in some other burrowing desert lizards, where individuals come up close to the surface in the early morning and only exit their burrows once they have reached an optimal temperature (Heatwole & Taylor, 1987), although surface temperatures are usually lowest during the hours just before dawn. Alternatively, skinks sitting at burrow entrances may have been foraging from the burrow entrance. *Tjakura* use both active and ambush foraging and may modify their strategy depending on cover, potentially using ambush foraging more where vegetation is sparse. The congeneric Slater's skink (*Liopholis slateri*) appears to rely on direct line of sight to forage effectively from burrow entrances (McKinney et al., 2014). For *tjakura* the abundance and type of prey available may also determine the foraging strategy used. Wildfire can temporarily reduce invertebrate abundance (Chapple, 2003), however, we did not measure prey abundance in our study or study very recently burnt burrows. Potential interactions between the foraging behaviour of *tjakura* and the abundance and composition of their prey following fire would be a useful further area of investigation.

### 4.2 Comments on methods and suggestions for further research

Our study demonstrates the value of camera trapping for monitoring the activity of *tjakura* and other reptile species that inhabit the same burrow for prolonged periods, especially where activity is concentrated in the vicinity of the burrow. Camera traps offer ethical and economical alternatives to live trapping and visual observation by facilitating the collection of large quantities of data captured unobtrusively (Bennett & Clements, 2014; Meek et al., 2014; Swann et al., 2011). They are well suited to studies of animal behaviour (Caravaggi et al., 2017) but still most often used in studies of mammals. Although reptiles can trigger infrared motion sensors when their body temperature differs substantially from ambient temperature, this is not a reliable method for detecting ectothermic animals with body temperatures that also frequently conform to surrounding temperatures (Bennett & Clements, 2014). We conducted a short pilot trial prior to our monitoring study, to test the efficacy of cameras set to take photographs every minute compared to cameras targeting the same burrow entrance and set to motion trigger. The time‐lapse approach was more effective and reliable in detecting lizard presence (i.e. captured more images) and enabled unbiased estimates of relative activity at different burrow entrances. However, with this approach, we were not able to determine what predators were doing at the burrows because there was no continuous record of predator behaviour. We recommend a combination of time lapse (primarily targeting *tjakura*) and motion triggered settings for similar studies in the future, as this would provide further opportunity to record mammalian and avian predators that may be active in the vicinity of the burrows.

Despite the excellent potential of cameras for monitoring burrowing species, effectively monitoring burrows with multiple entrances, and inhabited by multiple individuals is challenging. There has been little recorded about how *tjakura* use multiple entrances within a burrow system. Based on available information, we assumed that the main burrow entrance, identified by mound size and the proximity of the latrine (scat pile), would also be the most used entrance and therefore the most useful to monitor. However, in one of the two burrows where we monitored all available entrances, the skinks were more active at other entrances. These preliminary results suggest identifying a main entrance a priori for the purpose of monitoring *tjakura* activity could be unreliable. The use of burrow entrances is likely dynamic through time and may vary among individuals and depending on what the *tjakura* is doing. For example, Slater's skinks, also obligate burrow dwellers and closely related to *tjakura*, spend most of their time basking at a preferred (main) entrance of their burrows and use other entrances, which‐ever is in closest proximity, when returning from feeding or if the preferred entrance becomes shaded (Fenner et al., 2012). At the two burrows we monitored, all entrances were used by multiple individuals and some entrances (those in more open areas) were used more, or much more, than others. Other than vegetation cover, we did not observe any external features around highly used entrances that could assist in their a priori identification. Additional monitoring over longer periods and at more burrows would help to determine whether levels of relative usage among entrances within a burrow system remain stable through time, and whether the use of entrances varies based on the type of activity, for example whether a skink is foraging or basking.

Considering the variability in activity and vegetation cover at entrances within a burrow, it is likely that our ability to compare activity among entire burrow systems in relation to vegetation cover was constrained by only measuring activity at one entrance. Nevertheless, using proportional time active outside or inside a burrow entrance as a response variable has provided new insights about *tjakura* activity relative to vegetation, suggesting they may reduce activity outside the burrow when there is little cover. We recommend future research on *tjakura* behaviour includes monitoring multiple entrances as well as tracking the activity of individual skinks outside the burrow to determine home range, foraging behaviour and dispersal distances, to gain further understanding about the impact of vegetation cover on this species. At our study site, *tjakura* are a species of significant cultural importance to the local Anangu people. Local ranger groups and senior custodians of the region are working to protect the species. The tracking skills of some local Anangu, particularly older people, are exceptional, and a focussed tracking study would be extremely informative with respect to documenting the movements of *tjakura* away from the burrow and could potentially be combined with the use of radio tracking methods. Such a study should be designed by or codesigned with Anangu rangers and other community members and maximize opportunities for teaching younger generations these valuable skills.

## AUTHOR CONTRIBUTIONS

**Jenna C. H. Ridley:** Conceptualization (equal); data curation (lead); formal analysis (lead); investigation (lead); methodology (equal); project administration (supporting); writing – original draft (lead); writing – review and editing (equal). **Christine A. Schlesinger:** Conceptualization (equal); formal analysis (supporting); methodology (equal); project administration (lead); supervision (lead); writing – original draft (supporting); writing – review and editing (equal).

## FUNDING INFORMATION

There was no external funding for this research. The research was supported financially by Charles Darwin University, including Honours student funding allocation.

## CONFLICT OF INTEREST STATEMENT

The authors declare no conflict of interest.

## Data Availability

Data for this paper are available on Dryad https://doi.org/10.5061/dryad.vmcvdnczh.
